# Lineage-specific late pleistocene expansion of an endemic subtropical gossamer-wing damselfly, *Euphaea formosa*, in Taiwan

**DOI:** 10.1186/1471-2148-11-94

**Published:** 2011-04-12

**Authors:** Jen-Pan Huang, Chung-Ping Lin

**Affiliations:** 1 Department of Life Science & Center for Tropical Ecology and Biodiversity, Tunghai University, Taichung, Taiwan 40704; 2 Museum of Zoology, University of Michigan, 1109 Geddes Avenue, Ann Arbor, MI 48109, USA

## Abstract

**Background:**

Pleistocene glacial oscillations have significantly affected the historical population dynamics of temperate taxa. However, the general effects of recent climatic changes on the evolutionary history and genetic structure of extant subtropical species remain poorly understood. In the present study, phylogeographic and historical demographic analyses based on mitochondrial and nuclear DNA sequences were used. The aim was to investigate whether Pleistocene climatic cycles, paleo-drainages or mountain vicariance of Taiwan shaped the evolutionary diversification of a subtropical gossamer-wing damselfly, *Euphaea formosa*.

**Results:**

*E. formosa *populations originated in the middle Pleistocene period (0.3 Mya) and consisted of two evolutionarily independent lineages. It is likely that they derived from the Pleistocene paleo-drainages of northern and southern Minjiang, or alternatively by divergence within Taiwan. The ancestral North-central lineage colonized northwestern Taiwan first and maintained a slowly growing population throughout much of the early to middle Pleistocene period. The ancestral widespread lineage reached central-southern Taiwan and experienced a spatial and demographic expansion into eastern Taiwan. This expansion began approximately 30,000 years ago in the Holocene interglacial period. The ancestral southern expansion into eastern Taiwan indicates that the central mountain range (CMR) formed a barrier to east-west expansion. However, *E. formosa *populations in the three major biogeographic regions (East, South, and North-Central) exhibit no significant genetic partitions, suggesting that river drainages and mountains did not form strong geographical barriers against gene flow among extant populations.

**Conclusions:**

The present study implies that the antiquity of *E. formosa*'s colonization is associated with its high dispersal ability and larval tolerance to the late Pleistocene dry grasslands. The effect of late Pleistocene climatic changes on the subtropical damselfly's historical demography is lineage-specific, depending predominantly on its colonization history and geography. It is proposed that the Riss and Würm glaciations in the late Pleistocene period had a greater impact on the evolutionary diversification of subtropical insular species than the last glacial maximum (LGM).

## Background

Climatic oscillations of the Pleistocene period have significantly affected the population dynamics and genetic structures of extant taxa. The effects of glacial expansions and contractions on genetic characteristics and population demographics depend on the latitude, topography and life history of the species in question [1,2]. Genetic studies concerning temperate species in North America and Europe reveal a widespread pattern of northern expansion and southern refugia after the retreat of continental ice sheets [3]. In contrast, the tropics and sub-tropics had relatively stable climates during the Pleistocene period [4], which could have initiated different responses to glacial cycles and allowed inhabitants to generate genetic and demographic patterns distinct from those of temperate species [1,2]. The general effects of the Pleistocene ice ages on historical genetic structures of tropical and subtropical species remain largely unknown. However, several recent studies have demonstrated that tropical and subtropical species exhibit greater genetic diversity and limited post-glacial demographic expansion than temperate species [5,6].

**Figure 1:**
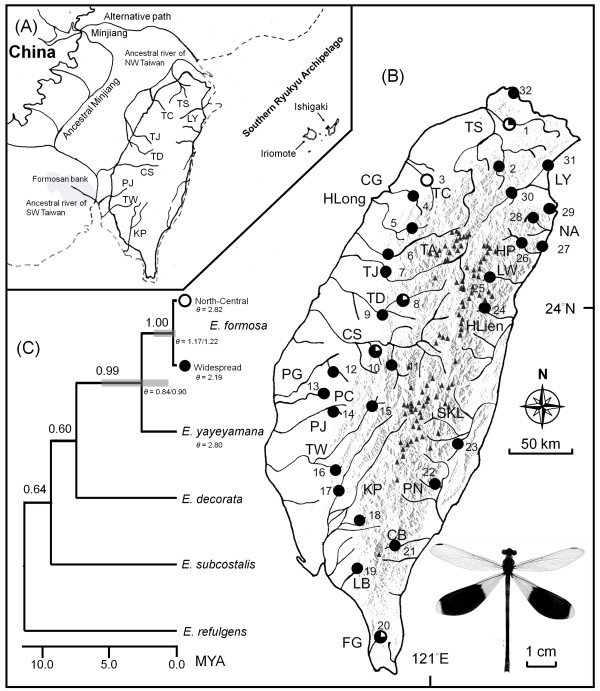
**Pleistocene river drainages of Taiwan, sampling localities and the species tree for *E. formosa***. (A). The proposed paleo-drainage systems of Minjiang on the continental shelf during the glacial period approximately 15,000 years ago [9]. (B) Present major river systems and mountain ranges of Taiwan, including the 32 sampling sites (in numbers) used in this study. The black triangles represent mountain peaks over 3,000 meters. (C) The species tree, divergence time and population size (*θ *= *N_e_μ*) of *E. formosa *jointly estimated with *COII *and *ITS *in *BEAST. Numbers above the branches represent the Bayesian Posterior Probability (BPP). The grey bars near the nodes are 95% HPD of divergence time estimates.

Taiwan is a subtropical island of approximately 36,000 km^2 ^located almost 100 km from the Asian mainland (Figure 1). The island was created by the collision of the Philippine Sea plate and Eurasian plate approximately 9 Mya [7], and uplifting processes thrust the proto-Taiwan above sea level approximately 5 Mya in the Pliocene [8]. Land bridges between Taiwan and the adjacent Asian continent formed and submerged intermittently during Pliocene and Pleistocene glacial cycles owing to changes in sea levels [9,10], resulting in Taiwan's fauna and flora having periodic contact with the Asian continent. Taiwan's central mountain range (CMR) reached its current altitude approximately 1 to 2 Mya, and currently includes more than 200 peaks exceeding 3,000 m [7,8]. The CMR transects the island from north to south, leaving a small portion of flat plains along the southwest coast (Figure 1B). The topographic complexity and high altitude of the CMR provided opportunities for diversification in Taiwan's endemic organisms [11,12].

A phylogeographic study of Taiwanese terrestrial organisms including higher plants (oak, *Cyclobalanopsis glauca *[13]; beech, *Castanopsis carlesii *[14]), vertebrates (bamboo viper, *Trimeresurus stejnegeri *[15]; rice frog, *Rana limnocharis *[16]) and invertebrates (common cricket, *Loxoblemmus appendicularis *[17]) demonstrates that the CMR is a prominent geographical barrier to species movement and gene flow among populations. The lowland to mid-elevation species in Taiwan exhibit a general pattern of eastern and western allopatric phylogroups separated by the CMR. However, several high-elevation small mammals including the mole-shrew (*Anourosorex yamashinai*) [18] and wood mouse (*Apodemus semotus*) [19] display distinct patterns of north-south phylogeographic sub-divisions. These divisions suggest that high mountain ranges could function as sky islands, providing interglacial refugia for high-altitude species, but genetic studies investigating strong dispersers demonstrate that the CMR is not an effective geographic barrier for vagile organisms such as the horseshoe bat (*Rhinolophus monoceros*) [20], montane bird (*Liocichla steerii*) [21] and wood spider (*Nephila pilipes*) [22], which generally exhibit high gene flow among homogeneous populations.

The Pleistocene river drainage plays a central role in shaping the biogeography and current genetic structures of Taiwanese freshwater fauna. Endemic freshwater vertebrates fall into three major biogeographic regions, the eastern, southern and north-central regions, reflecting the paleo-drainages of Pleistocene land bridges [23] (Figure 1A). Previous research concerning freshwater organisms including minnows (*Zacco pachycephalus*) [24], ray-finned fish (*Varicorhinus barbatulus*) [25] and stream crabs (*Candidiopotamon rathbunae*) [26] reveals substantial genetic differentiation among populations that primarily correspond to these paleo-drainages. Studies investigating low-elevation wetland fishes suggest that low genetic divergence among populations [27] could result from seasonal flooding or periodic connections to ancestral river drainages. Early studies indicate that the genetic structure of freshwater fauna with strict aquatic life histories is primarily the result of interplay between vicariance caused by ancient river drainages and the organism's vagility. However, a phylogeographic study concerning the predominant and diverse aquatic insects in Taiwan's freshwater ecosystems have not been conducted.

Aquatic insects have a complex life history comprising an aquatic immature stage constrained by freshwater habitats, and a winged adult stage with dispersal being limited by terrestrial barriers and flying ability [28]. The dispersal abilities of aquatic insects vary greatly among taxa and according to age and sex, ranging from strong dispersers such as dragonflies to less vagile mosquitoes [29]. Determining whether aquatic insects were affected by the same isolating barriers that generated genetic structure in terrestrial and freshwater fauna is a prerequisite for understanding the relative impact of river drainage and mountain vicariance in Taiwan. *Euphaea formosa *Hagen, 1869 (Insecta: Odonata), is a gossamer-wing damselfly species endemic to Taiwan, found throughout the island in the lowlands and fast flowing mountain streams at an altitude of less lower than 1,500 meters. The dispersal ability of adult *E. formosa *has not been estimated but observations from mark-recapture studies suggest that adult dispersal could be high (>10 km). Therefore, *E. formosa *is a suitable freshwater insect candidate that should have developed the same genetic signatures observed in more vagile terrestrial species in Taiwan [20-22].

The present study uses DNA sequences of mitochondrial cytochrome oxidase II (*COII*) and nuclear internal transcribed spacer (*ITS*) to investigate the phylogeography and demographic history of *E. formosa*. The aim was to determine whether this endemic damselfly exhibits phylogeographic patterns and a genetic structure similar to terrestrial and freshwater fauna in Taiwan. Furthermore, the historical demography and lineage divergence time of *E. formosa *was investigated to determine the concordance between the population history and major climatic cycles during the Pleistocene period.

## Results

### Sequence variation

A total of 500 bp of mitochondrial *COII *sequences were obtained from 159 individuals. The samples included 51 unique haplotypes in *E. formosa *and four in *E. yayeyamana*. The nuclear *ITS *sequence contained 681 bps including 100 unique haplotypes in 125 *E. formosa *and two in *E. yayeyamana*. The AT content was 71% for *COII *but *ITS *demonstrated no bias towards A-T rich sequences (46%). The pairwise sequence divergence of *COII *ranged from 0 to 8.2% within *E. formosa*, and from 4.8 to 9.4% between *E. formosa *and *E. yayeyamana*. For *ITS*, the pairwise sequence divergence ranged from 0 to 1.7% within *E. formosa*, and from 1.7 to 3.2% between *E. formosa *and *E. yayeyamana*. A microsatellite locus with GA repeats (0-8) appeared in the *ITS1 *region. The estimated *ω *ratio of *COII *sequences (0.021 in M0) was much less than one, and the LRT of M1 vs. M2 was insignificant (*χ*^2 ^= 0.046, *p *= 0.977, df = 2). These results indicate that the M2 selection model did not fit the data significantly better than the M1 neutral model. Therefore, the neutral evolution of *COII *nucleotide sites in *Euphaea *damselflies is possible.

### Gene Trees and Species Phylogeny

**Figure 2:**
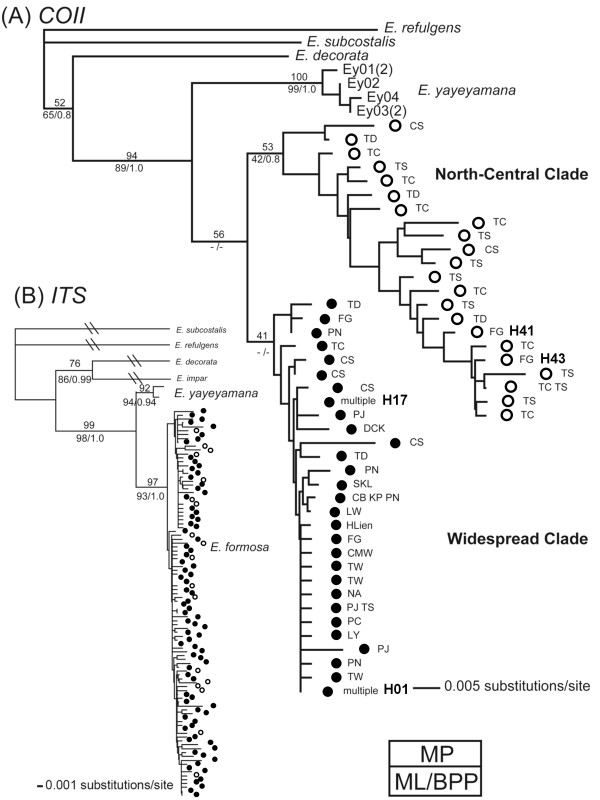
**Phylogenetic relationships between *E. formosa *and out groups**. (A) *COII *trees (lnL=-2276). (B) *ITS *trees (lnL=-3225). Numbers near the nodes are support values of the Maximum Parsimony (MP) bootstrap (above), Maximum Likelihood (ML) bootstrap (lower left) and Bayesian Posterior Probability (BPP) (lower right). Nodes without support values have values below 50%. Abbreviations of the haplotypes and sampling localities are listed in Additional file 1.

The HKY+I+Γ (lnL = -2276) and TVMef+I (lnL = -3225) models were selected as the best-fitting models concerning sequence evolution of *COII *and *ITS*, respectively. The *COII *phylogeny was well resolved with strong support at the species level, suggesting a monophyletic *E. formosa *with respect to sister *E. yayeyamana *(Figure 2A). This phylogeny identified two distinct *COII *haplotype clades (North-central & widespread) separated by a deep phylogenetic split. The North-central clade was restricted to northern and central Taiwan, with the exception of two derived southern haplotypes (H41 & H43), suggesting recent long-range dispersal. This clade had a balanced tree topology, suggesting a relatively large population that had been stable for a long time period. In contrast, the widespread clade contained two high frequency haplotypes (H01 & H17) that were widely distributed throughout the island. The widespread clade had a star-like phylogeny, indicating rapid population expansion. The *ITS *tree suggested a monophyletic *E. formosa *sister to *E. yayeyamana *(Figure 2B). This tree presented with a shallow topology and revealed no apparent population sub-structuring within *E. formosa*. The species tree estimated with *BEAST recovered *E. formosa *as a reciprocally monophyletic group, with *E. yayeyamana *being a sister species (Figure 1C). Within *E. formosa*, the haplotypes were grouped into the North-central and widespread clades, but the relationships among the sampled populations within these two clades were not resolved. This indicates a high number of shared haplotypes among the populations of both North-central and widespread clades.

### Population genetics

The haplotype diversities (*h*) of *COII *ranged from 0 to 1, and the haplotype diversities of *ITS *ranged from 0.75 to 1 (Additional file 1). The nucleotide diversities (*π*) of these two gene regions varied between 0 and 0.028 for *COII*, and between 0.002 and 0.009 for *ITS*. Populations with the highest *COII *nucleotide diversities, representing ancestral sources of extant *E. formosa*, were present in five major river systems in northern and central Taiwan (TSKL, 0.026; TC, 0.028; TDtk, 0.023; CSlhc, 0.023; FG, 0.026). These populations corresponded to the two proposed paleo-drainage systems of Taiwan, with the exception of the southern population from the Fongkang river (FG) (Figure 1A). However, the populations from eastern Taiwan (site 21 to 31) and the three river systems of the proposed paleo-drainage in the southwestern plains (PJ, TW & KP), exhibited low *COII *nucleotide diversities (0.001-0.008), indicating recent origins or population bottlenecks. The relatively high *h *(0.657) and low *π *(0.003) values within the widespread *COII *clade suggested rapid population growth from a small ancestral population (Table 1). The North-central *COII *clade, which had high values for *h *(0.996) and *π *(0.021), indicated a stable population with a long-term, large population size or admixed populations from separated ancestral lineages. However, *ITS *exhibited high haplotype diversities and low nucleotide diversities in all populations, suggesting population expansion. Population differentiation (*F*_ST_) was higher in the North-central clade than the widespread *COII *clade (Table 1). The number of individuals estimated to be long-term effective migrants (*Nm*) in the widespread *COII *clade was greater than that of the North-central *COII *clade and *ITS *estimation. Tajima's *D *of the widespread *COII *clade and *ITS *were negative and significantly different to zero, suggesting past population expansions. Tajima's *D *of the North-central *COII *clade was positive, but not significantly different to zero. Fu's *F*_S _calculated for the widespread, North-central *COII *clade and *ITS *were negative and significantly different from zero, implying past population growth. The AMOVA of *COII *indicated non-significant (8% of molecular variance) genetic differentiation between east and west groups and among east, south, and North-central groups (Table 2). Significant population differentiation (P < 0.0001) was evident among populations within groups (24-26%) and within populations (67%). The majority of *ITS *variance was evident within populations (97%). No significant correlation was detected between pairwise genetic and geographic distances of the widespread *COII *clade (*r *= -0.078, *P *= 0.923), whereas significantly positive correlations between pairwise genetic and geographic were present in the North-central *COII *clade and *ITS *(*r *= 0.637 and 0.103, *P *= 0.073 and 0.061, respectively).

**Table 1:** Population genetic statistics among *E. formosa *damselfly populations of the major phylogenetic lineages and all samples combined.

Clade	n	nh	*h*	*π*	*D*	*F*_S_	*Nm*	*F*_ST_
*COII *Widespread	136	28	0.657	0.003	-2.465***	-28.441***	12.96	0.037
North-Central	23	22	0.996	0.021	0.450	-11.974**	9.93	0.051
Total	159	51	0.754	0.015	-1.172	-20.191***	0.73	0.407
*ITS*	122	82	0.962	0.005	-2.717***	-24.592***	9.26	0.026

n, sample size; nh, number of haplotype; *h*, haplotype diversity; *π*, nucleotide diversity; *D*, Tajima's *D*; *F*_S_, Fu's *F*_S_; *Nm*, number of effective migrants; *F*_ST_, fixation index; **P < 0.001; ***P < 0.0001.

**Table 2:** Analysis of molecular variance (AMOVA) with distribution of genetic variation among populations of *E. formosa*.

Gene	Source of variation	**d.f**.	Sum of squares	% of variation	Fixation index (Φ)
*COII*	Among groups (East & West)	1	28.290	7.65	0.07648
	Among populations Within groups	30	237.639	25.92	0.28061***
	Within populations	135	354.855	66.44	0.33563***
	
	Among groups (East, South, & North-Central)	2	50.293	8.42	0.08421
	Among populations Within groups	29	215.637	24.10	0.26311***
	Within populations	135	354.855	67.48	0.32517***

*ITS*	Among groups (East & West)	1	2.443	-1.06	-0.01061
	Among populations Within groups	29	134.604	3.21	0.03174
	Within populations	94	385.785	97.85	0.02146
	
	Among groups (East, South, & North-Central)	2	8.882	-0.13	-0.00128
	Among populations Within groups	28	128.165	2.83	0.02827
	Within populations	94	385.785	97.30	0.02703

***P < 0.0001

### Demographic history and coalescence time

**Figure 3:**
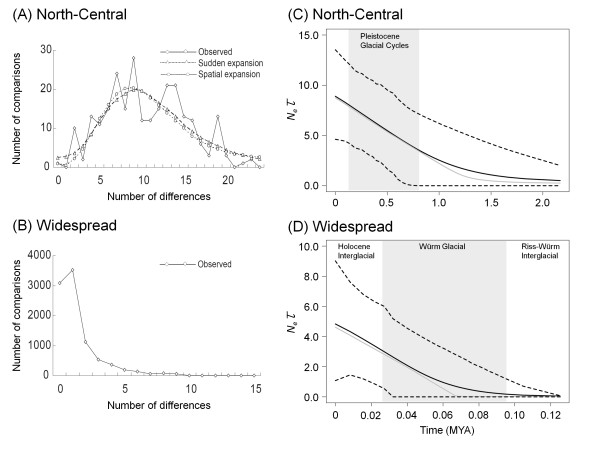
**Mismatch distributions and Bayesian demographic analyses of *E. formosa COII *sequence data**. (A) The North-central clade has a multimodal distribution that differs from sudden demographic and spatial expansion models. (B) The widespread clade exhibits a distinct unimodal distribution. (C) The EBSP of the North-central clade demonstrates relatively stationary populations between 0.6 and 2 Mys, with a demographic expansion over much of the Pleistocene glacial cycles. (D) The EBSP of the widespread clade indicates a population growth beginning near the end of the glacial Würm period (0.03 Mya) and into the Holocene interglacial period.

The North-central *COII *clade exhibited a multimodal mismatch distribution, indicating a relatively stable population throughout time. This finding differs significantly from the simulation of sudden expansion and spatial expansion models (SSD = 0.0084 and 0.0086, *P *= 0.314 and 0.268; *r *= 0.0197 and 0.0197, *P *= 0.199 and 0.197) (Figure 3A). The widespread COII clade exhibited a smooth unimodal distribution, suggesting past population expansions (Figure 3B). However, this mismatch distribution did not converge with the expected sudden demographic and spatial expansion models using the least square procedure. The EBSP of the North-central *COII *clade revealed a slowly growing curve (Figure 3C). These results suggest these were relatively stable populations during the past 1.5 million years, with an approximate two fold increase in population sizes from 4.82 to 8.90, beginning approximately 0.6 Mya and during the late Pleistocene glacial cycles (EBSP 95% HPD of *N_e_*τ = 0.66-8.66 and 4.62-13.53, respectively) (Figure 3C). For the widespread *COII *clade, the EBSP with 95% HPD demonstrated that the population underwent population growth approximately 0.03 Mya, towards the end of glacial Würm MIS6 in the Riss period, with an estimated effective population size of 2.33 (EBSP 95% HPD of *N_e_*τ = 0.01-4.84) (Figure 3D). During the interglacial Holocene period, the populations increased more than two-fold, reaching a size of 4.84 (EBSP 95% HPD of *N_e_*τ = 1.08-9.02). The estimated *T_mrca _*for *E. formosa *and sister species *E. yayeyamana *was approximately 2.6 Mya in the late Pliocene (mean = 2.586, 95% CI = 0.483-6.216, ESS = 1298). The *T_mrca _*for the origin of *E. formosa *was approximately 0.3 Mya in the middle Pleistocene period (mean = 0.319, 95% CI = 0.127-1.752, ESS = 1086).

## Discussion

### Evolutionary history of *Euphaea formosa*

This study provides the first phylogeographic analysis of endemic freshwater insects in Taiwan. The phylogenetic results suggest that the common ancestors of *E. formosa *and its sister species *E. yayeyamana *of the southern Ryukyu Archipelago, began diverging approximately 2.6 Mya during the late Pliocene (Figure 1C), approximately 2.5 Mya after the emergence of Taiwan above sea level (4-5 Mya [8]). During the Pleistocene glaciations, the continental shelf under the East China Sea emerged and provided land bridge connections between the southern Ryukyus Archipelago and the Asian continent [9,10]. These land bridge connections allowed ancestral *Euphaea *lineages from the Asian mainland to disperse and colonize Taiwan, Ishigaki and Iriomote Island. The subsequent vicariant isolation and divergence between Taiwan and the southern Ryukyu Archipelago is likely to have initiated diversification between these two insular species during the Pleistocene glacial cycles. In addition to vicariant isolation, ecological adaptation to the greater availability of larval prey in Taiwan compared with the Ishigaki and Iriomote islands is another probable factor contributing to the larger body size of *E. formosa *compared with *E. yayeyamana *[30]. With the exception of size, no morphological characteristics distinguish between these two damselflies, and the molecular phylogenies in this study clearly demonstrate that *E. formosa *and *E. yayeyamana *are monophyletic sister species with average genetic distances of 8% (*COII*) and 2.5% (*ITS*). Therefore, these two insular gossamer-wings are distinctive "genetic" and "phylogenetic" species [31].

The mitochondrial *COII *and nuclear *ITS *gene trees for *E. formosa *were not fully congruent. In addition to incongruence associated with the gene tree/species tree, the results suggested a possible introgression or incomplete sorting of lineages in the ancestral polymorphism of these two markers. The jointly estimated coalescence time for *COII *and *ITS *suggests that the extant *E. formosa *populations probably originated approximately 0.3 Mya, during the middle Pleistocene period. The existence of a deep phylogenetic split and substantial genetic differentiation between the North-central and widespread *COII *clades, together with the monophyly of haplotypes from these two lineages, imply that there were at least two periods of colonization on the island. Alternatively, there was a single colonization followed by divergence of the two clades within Taiwan (Figure 1&2). These two evolutionary scenarios are equally plausible for the origin of extant *E. formosa*, where the ancestral *E. formosa *colonized northwestern Taiwan via the emerging land bridges and rivers connecting it to the Asian continent as a result of lowered sea levels during repeated glacial periods approximately 0.3 Mya during the middle Pleistocene period [9,10].

Previous estimates of colonizing periods for Taiwanese freshwater fishes are less than one Mya, falling within the time frame of the middle to late Pleistocene. These estimates include minnows (0.06 Mya [24]), cyprinid fish (0.11-0.39 & 0.1-1 Mya [32,33]) and goby (0.87 Mya [34]), but the colonizing period for spiny loach is estimated to be the middle to late Pliocene period (2.22-2.75 & 3.41-4.23 Mya [35]). In contrast, several studies concerning Taiwanese lowland terrestrial animals estimate the colonization periods to be greater than two Mya, ranging from the Pliocene to early Pleistocene periods (bamboo viper, 2-3 Mya [15]; lizard, 5-8 Mya [36]; crab, 5-7 Mya [26]; common cricket, 1.8 Mya [17]). Assuming an average mitochondrial *COII *clock for the insects (1.15% divergence/lineage/myrs [37]) and a scaled *ITS *clock, the estimate based on the current study is that ancestral *E. formosa *first colonized the island during the early to middle Pleistocene period (0.13-1.8 Mya), preceding the colonizing periods for the majority of Taiwanese freshwater fish, which have low migratory capabilities limited by availability of continuous aquatic habitats. Therefore, *E. formosa*, a vagile aquatic insect present in lowland rivers and streams, exhibits an ancient colonization history older than most freshwater vertebrates but younger than lowland terrestrial animals.

In addition to the apparently higher dispersal ability assisted by winged adults, an explanation for the antiquity of *E. formosa*'s colonization could be the past climate and vegetations in the continental land bridges during the Pleistocene glaciations. Paleo-environmental and palynological studies suggest that the climate of exposed land bridges connecting the Asian continent to lowland Taiwan during extended glaciations were cooler and dryer than at present, and the vegetation zones in these continental land bridges were predominantly grasslands [38,39]. Dry grassland habitats with limited river systems and frequently interrupted water supplies caused by weaker monsoons acted as migratory barriers for freshwater fish, but were relatively weak barriers for *Euphaea *damselflies, particularly at the larval stage. *Euphaea formosa *are damselflies of the only two odonate families known to possess lateral abdominal gill appendages in addition to the usual three caudal gills (the Oriental-Palearctic Euphaeidae & Neotropical Polythoridae [40]). Lateral abdominal gills were considered an adaptation to riverine habitats subject to intermittent oxygen deficiency [40], which would allow heavy sclerotized larvae to take refuge in the hyporheal zone where oxygen concentration is low during dry seasons [41,42]. The ability of *E. formosa *larvae to survive in periodically dry streambeds, which probably characterized the river systems of the Pleistocene dry grasslands, could facilitate an early colonizing history into the lowlands of Taiwan compared with other freshwater fish.

### Phylogeography and lineage-specific population expansion

The AMOVA indicated no significant genetic differentiation in mitochondrial *COII *or nuclear *ITS *between east and west groups or among east, south and North-central groups (Table 2). Therefore, it is unlikely that river drainages or the CMR constituted a strong physical barrier to current gene flow among populations. However, substantial genetic differentiation appears among populations within regions. These results suggest that *E. formosa *populations are not panmictic, and that current gene flow among populations is likely to be restricted by geographic distances. Nevertheless, Pleistocene river drainages and the CMR played major roles in shaping the colonization and diversification history of *E. formosa*. Genetic analyses suggested that extant *E. formosa *populations could be derived from two independent ancestral source lineages due to past allopatric fragmentation, which reflected the Pleistocene river drainages of continental land bridges. In this scenario, the North-central clade (north of Choshui River) likely derived from the paleo-drainages of northern Minjiang and adjacent northwestern Taiwan first colonized northern Taiwan early during the Pleistocene period (Figure 1&2). Later, the ancestral widespread clade, which originated from the paleo-drainages of southern Minjiang, reached central-southern Taiwan in the middle of the Pleistocene period. These interpretations have been supported by the findings that *E. formosa *populations with the highest genetic diversity were distributed between five major river systems corresponding to these paleo-drainages [9,23] (Additional file 1). An alternative scenario concerning postglacial re-colonization from two previously isolated refugia is equally likely to generate the genetic divergence between the North-central and widespread clades. The land bridges connecting the Asian continent with the island of Taiwan have been proposed as glacial refugia for lowland freshwater species such as endemic minnows and cyprinid fish [9,24,33]. The average temperature during the Pleistocene glaciations in East Asia was at least 5°C lower than at present [38,39]. Therefore, during the glacial maxima the distribution of *E. formosa *would have been restricted to the edge of the island and the exposed continental land bridges. The paleo-drainages of northwestern Taiwan and southern Minjiang could have acted as isolated glacial refugia for ancestral *E. formosa *populations. As a warmer climate and more favorable habitats appeared during the Pleistocene inter-glaciations, *E. formosa *repopulated the lowlands of western Taiwan from the two refugia.

The phylogeographic and historical demographic analysis carried out in this study indicated that the effect of Pleistocene climatic changes on the population dynamics of *E. formosa *were lineage-specific and depended predominantly on the colonization history and geography of the two evolutionarily independent clades. The haplotype diversity of *COII *was higher in northwestern populations than populations in the south, with the most eastern Taiwan populations having only the widespread haplotypes H01 and H17. This pattern of haplotype distribution indicates that the founders of the eastern populations originated from western Taiwan and moved to southern regions of the island. This southern dispersal route into eastern Taiwan is evident in an endemic cyprinid fish [33] and the common cricket [17]. In addition to the historical range expansion, this study provides clear evidence that the widespread clade experienced a demographic expansion approximately 0.03 Mya during the late Pleistocene period. The onset of this demographic expansion was associated with the Holocene interglacial period, when the warmer climate and available lowland streams on the eastern side of the CMR could have permitted the damselfly to colonize and expand into eastern Taiwan. In contrast, the North-central clade maintained a slowly growing population size throughout much of the early and middle Pleistocene periods, possibly because of the stable aquatic habitats of northern Taiwan. Northern Taiwan experiences northeast monsoons during the winter (dry seasons) and has much higher precipitation than central and southwestern Taiwan [43], and the water levels of these northern rivers and streams are stable throughout the year and are capable of sustaining constant population sizes and maintaining high genetic diversity. In contrast, the widespread clade of the central and southern rivers experienced more frequent seasonal droughts during winter-spring dry seasons, and consequently generated smaller population sizes and decreased genetic diversity due to genetic drift in fluctuating populations. The rivers and streams in eastern Taiwan are much shorter than those of western Taiwan owing to the steep slopes of the eastern CMR. Therefore, they support smaller populations with lower genetic diversity.

The historical demography of extant species was affected by climatic cycles during the Pleistocene period [2,3]. Phylogeographic studies concerning European and North American fauna suggest that the last glacial maximum (LGM, approximately 0.018 Mya) was a major climatic event structuring the historical population dynamics of temperate taxa [2,3]. After the retreat of the LGM, temperate species frequently expanded from southern refugia into previously glaciated northern ranges. However, the findings of this study indicate that the onset of demographic expansion in subtropical *E. formosa *predated the LGM period, occurring during the middle or late Pleistocene period (0.03 and 0.6 Mya). Recent research investigating an endemic babbler in East Asia suggests a similar late Pleistocene population growth (0.17 Mya), after release from separated glacial refugia around southeast China [44]. *E. formosa *demonstrates a less drastic demographic expansion trend in terms of magnitude of population growth than many temperate taxa [1-3]. Therefore, a post-LGM population expansion model that characterizes several temperate species cannot describe the Pleistocene population dynamics of subtropical lowland taxa such as *E. formosa*. These results imply that earlier glacial periods such as Riss and Würm during the late Pleistocene period could have had greater impact on the historical demography and levels of genetic diversity in subtropical and tropical species than previously thought. Tropical species are often phylogeographically older than their temperate counterparts, and are likely to experience earlier climatic oscillations in the Pleistocene period [5]. A relatively mild, late Pleistocene climate in subtropical East Asia [4] and available glacial refugia in continental land bridges [9] could have alleviated demographic stresses during the Pleistocene glaciations, which in turn generated relatively modest population fluctuations in *E. formosa*.

## Conclusions

This study has investigated Pleistocene climatic, geographic and natural history factors underlying the evolutionary diversification of an aquatic insect in Taiwan. The endemic gossamer-wing damselfly originated during the early to middle Pleistocene period, earlier than the late Pleistocene colonization of the majority of Taiwanese freshwater vertebrates. The antiquity and colonization history of *E. formosa *could be due to the flying ability of the adult and larval tolerance to the periodically dry grassland habitats that characterized Pleistocene land bridges. The extant *E. formosa *population consists of two phylogenetically independent lineages that either originated from the Pleistocene river drainages of northern and southern Minjiang or re-colonized from two separated refugia. Each lineage has undergone divergent evolutionary trajectories within the island. The present findings revealed the spatial and demographic expansion of the widespread lineage that can be dated to the late Pleistocene period (approximately 30,000 years ago). This population expansion was probably due to the availability of habitats in eastern Taiwan during the warmer Holocene interglacial period. In contrast, the North-central lineage has maintained a relatively stable population during the past one and half million years, possibly because of the stable aquatic habitats of northern Taiwan. The ancestral *E. formosa *expanded into eastern Taiwan via a southern dispersal route, indicating that the CMR formed a barrier to the east-west dispersal of this damselfly. However, the CMR and Pleistocene river drainages are not strong geographic barriers to current gene flow among *E. formosa *populations, with genetic structures being shaped by geographic distances within the three biogeographic regions. This study led to the proposal that the Riss and Würm glaciations in the late Pleistocene period had a greater impact on the evolutionary diversification of subtropical insular species than the LGM. Further studies investigating taxa with diverse life history characteristics could elucidate the general effect of recent climatic oscillations on the evolutionary diversification of subtropical species.

## Methods

### Biology of *E. formosa *and sampling

Male *E. formosa *damselflies are characterized by black bands on the hind wings and distinct red stripes on the thorax (Figure 1). Adults occur on the wing from April to November, with a population peak between June and August. They appear near streams with rapid water flow and an open canopy. Mature males with established territories usually perch on rocks or plants, and exhibit aggressive territorial behavior towards intruding con-specific males. Females appear periodically inside these territories and mate with territory owners.

One hundred and fifty nine *E. formosa *individuals from 32 sites, representing 27 major rivers and streams throughout Taiwan, were sampled (Figure 1B, Additional file 1). Voucher specimens were preserved in 95% ethanol and stored at -80°C in Tunghai University. For outgroups, five *Euphaea *species from Southeast Asia including *E. decorata*, *E. impar*, *E. refulgens*, *E. subcostalis*, and *E. yayeyamana*, which are endemic to the Ishigaki and Iriomote Islands of the Ryukyu Archipelago situated 200 km east of Taiwan, were used. The *ITS *sequence of *E. impar *was obtained from GenBank (AJ746322). An earlier study comparing external morphological characters of *E. formosa *and *E. yayeyamana *demonstrated no distinct differentiation, except that *E. yayeyamana *is smaller (insular dwarfism) [30]. This suggests a close phylogenetic relationship between these two species. To determine the taxonomic status and the degree of genetic differentiation between these *Euphaea *species, six *E. yayeyamana *individuals were used for the analyses.

### DNA extraction, sequencing, and alignment

DNA was extracted from insect thoracic muscle using the standard CTAB, pheno-chloroform protocol, followed by ethanol precipitation [45]. The primers C2-J-3102 [46] and *Euphaea*-specific C2-N-3740 (5'-TCA TCT AGT GAG GCT TCA-3') were used to amplify the *COII *gene. A eukaryote-specific 18SF/28SR primer set was used to amplify *ITS1*, *ITS2 *and *5.8S *genes [47]. PCR reactions were performed in an Ependorf thermocycler (*Mastercycler Gradient*, Hamburg, Germany). PCR reactions contained a total volume of 50 μl, composed of 1 μl of ProTaq polymerase (2 u/μl, Protech Technology, Taiwan), 2 μl of 10 mM of each primer, 4 μl of 1 mM dNTPs, 5 μl of ProTaq buffer, 35 μl of ddH_2_O and 1 μl of extracted DNA (100 to 400 ng/μl). For *COII*, the PCR profile was as follows: denaturing at 94°C for one minute, 35 cycles of amplification at 94°C for one minute followed by 53°C for one minute and 72°C for one minute, and a final extension at 72°C for 10 minutes. For *ITS*, the PCR profile was the same as that for *COII *except the annealing temperature was 52°C. PCR products were purified using a Gel/PCR DNA Fragments Extraction Kit (Geneaid, Taipei, Taiwan) and cloned into competent cells (dH-5α) using the T&A cloning kit (RBC, Taipei, Taiwan). Positive clones were confirmed by performing PCR reactions using M13F/M13R primers. Plasmid DNA was sequenced in both directions on an ABI PRISM™ 377 automatic sequencer (Perkin Elmer, USA) at the Mission Biotech, Taiwan. Manually inspected and edited DNA sequences from raw chromatograph data were aligned using the Clustal W method in MegAlign (DNAStar package, Madison, USA). *COII *sequences were translated into amino acid sequences using a mitochondrial genetic code of *Drosophila *in MacClade v.4.06 [48] to check for possible stop codons caused by ambiguous sequencing. Thirteen paralogous *ITS2 *sequences with large indels (20 to 58 bps) were excluded from the analyses. Sequences used in this study were deposited in GenBank (EU603519-EU603681). The sequence alignment and associated phylogenetic trees were submitted to the TreeBASE http://purl.org/phylo/treebase/phylows/study/TB2:S11020.

### Phylogenetic analyses

Phylogenetic analyses were performed using Maximum Parsimony (MP) in PAUP* v.4.0b10 [49]. Parsimony branch supports were calculated using bootstrapping of 1,000 replicates of tree bisection and reconnection (TBR) branch swapping, with 10 replications of random sequence addition. For Maximum Likelihood (ML) and Bayesian phylogenetic analyses, the best-fitting model of nucleotide substitution was selected in MODELTEST v.3.7 [50] using the Bayesian Information Criterion (BIC). ML branch supports were calculated with 100 bootstrap replications in the PhyML 3.0 web sever [51]. Bayesian analyses were carried out using MrBayes v.3.1.2 [52], with prior settings and the parameters of nucleotide substitution models being estimated using MODELTEST. Bayesian posterior probabilities (BPP) were calculated separately for each gene partition. Two separate Bayesian runs, each with four Markov chains, were performed simultaneously. The Markov Chain Monte Carlo (MCMC) searches were run for 1 × 10^7 ^and 6 × 10^6 ^generations for *COII *and *ITS*, respectively, with trees being sampled for every 100 generations. MCMC runs were terminated after the average split frequencies went below 0.01 and the Convergence Diagnostic Potential Scale Reduction Factor (PSRF) approached one, suggesting convergence of the two separate runs [53]. One quarter of the sampled trees (25,000 and 15,000 for *COII *and *ITS *respectively) were discarded as burn-in. The remaining trees were imported into PAUP* for constructing a 50% majority consensus tree. A species tree was co-estimated from *COII *and *ITS *gene trees using *BEAST [54] implemented in BEAST v.1.6.1 [55]. The substitution model, clock model and tree model of the two genes were set as unlinked. The mutation rate of *COII *was set to the average value found in arthropods (1.15% divergence/lineage/myrs [37]), and the mutation rate of *ITS *(0.49%, 95% PD = 0.00248-0.00727, ESS = 362) was estimated relative to *COII*. The relaxed local clock model was used, with estimated rate change counts of 2.0 and 1.3 for *COII *(95% PD = 1-4, ESS = 1200) and *ITS *(95% PD = 0-3, ESS = 4743), respectively. The species tree prior was set to the default option of Yule process. The MCMC analyses were run for 5 × 10^8 ^generations, with parameters sampled for every 1 × 10^4 ^generations and the first 10% of the runs being discarded as burn-in. The convergence of runs was determined by examining likelihood scores through time plot using TRACER v. 1.5 [55].

### Neutrality and population genetic analyses

A codeml module of PAML (v. 4, [56]) was used to detect the signature of natural selection at *COII*. The non-synonymous versus synonymous substitution ratio (*ω*: *d*_N_/*d*_S_) was calculated under model M0 (no site rate heterogeneity) with likelihood tree topologies obtained from the phylogenetic analyses. The Likelihood Ratio Test (LRT) was used to detect the significance between model M1 (nearly neutral) and model M2 (selection) using chi-square statistics. Haplotype diversity (*h*), nuclear diversity (*π*), exact tests of population differentiation, pairwise and overall *F*_ST _among populations were calculated using DnaSP v.4.0 [57]. Mantel tests were used to examine the association between the genetic (*F*_ST_) and geographic distance (km) of populations using 10,000 permutations and a Reduced Major Axis (RMA) regression in IBD [58]. The population genetic structures were tested with the Analysis of Molecular Variance (AMOVA) with 1,000 permutations in ARLEQUIN v.3.01 [59]. Populations were assigned to two (east or west) and three groups (east, south, or north-central) to test for restricted gene flow among biogeographic regions identified in earlier studies. Three hierarchical levels of genetic variance including within populations (Φ_ST_), among populations within groups (Φ_SG_) and among groups (Φ_GT_) were calculated. Tajima's *D *and Fu's *F*_S _were computed using ARLEQUIN with 1,000 permutations. Positive values of Tajima's *D *and Fu's *F*_S _indicated possible balancing selection (single locus) or population subdivision (multiple loci), whereas negative values suggested positive selection (single locus) or expansion of population size (multiple loci).

### Demographic history and divergence time

The demographic history was inferred from non-recombined *COII *haplotypes using a mismatch distribution analysis with 1,000 bootstrap replications in ARLEQUIN. The analyses were carried out separately for the North-central and widespread clades of *COII *because of a deep phylogenetic gap between these two lineages (see phylogeny result below). The goodness of fit between observed data and expected demographic models was estimated using the Sum of Square Deviations (SSD). The Harpending's Raggedness index (*r*) was calculated with 1,000 permutations to test the significance of *r *between the observed and simulated distributions. A Bayesian coalescence-based framework [60] was employed to infer the demographic history and lineage divergence time in *E. formosa *on the basis of both loci. We computed the posterior distributions of effective population sizes (*N_e_*τ), time to the most recent common ancestor (*T_mrca_*) and the multi-locus Extended Bayesian Skyline Plot (EBSP) [61] using a linear model implemented in BEAST v.1.6.1 [55]. *COII *and *ITS *were set as unlinked while assuming the same demographic history. The mutation rate setting was the same as in the above *BEAST analyses. The starting population size was set to one, and the scale factor *k *was set to two. Both gene trees were reconstructed using an uncorrelated relaxed lognormal clock model. The window size for *COII *and *ITS *random walk integer was set to two and eight, respectively. The MCMC searches were run for 1 × 10^8 ^generations with parameters sampled for every 1 × 10^4 ^generations and the first 10% of the runs being discarded as burn-in. Each analysis was repeated several times to optimize the scale factors until there were no suggesting messages in the log file. The Effective Sample Size (ESS) of estimated parameter values was determined using Tracer v. 1.5 [55], and the EBSPs were visualized using R (v. 2.12.0; http://www.r-project.org/).

## Authors' contributions

CPL designed the study; JPH and CPL carried out the field work; JPH generated the molecular data and conducted all DNA sequence and statistical analyses with input from CPL; CPL and JPH wrote the manuscript. The authors read and approved the final manuscript.

## Supplementary Material

Additional file 1 **Specimen and sequence data**. Collecting locality of *E. formosa *and outgroup specimens, and the summary statistics of the sampled damselfly populations. Click here for file
