# Supplementary material for: Lineage-specific late pleistocene expansion of an endemic subtropical gossamer-wing damselfly, Euphaea formosa, in Taiwan
Source: BMC Evol Biol. 2011 Apr 12;11:94. doi: 10.1186/1471-2148-11-94 (PMC3094233; doi:10.1186/1471-2148-11-94)
Supplement: Additional file 1 — Specimen and sequence data. Collecting locality of E. formosa and outgroup specimens, and the summary statistics of the sampled damselfly populations. [file 1471-2148-11-94-S1.DOC]

| Species | Localities | Abbr. | *COII* Haplotypes | # | *h* | *π* | *ITS* Haplotypes | # | *h* | *π* | GPS Coordinates |
| --- | --- | --- | --- | --- | --- | --- | --- | --- | --- | --- | --- |
| *E. formosa* | **Danshui** | TS |  |  |  |  |  |  |  |  |  |
|  | Nanshih | TSNS | H01(4), H06 | 5 | 0.4 | 0.00080 | I32, I56, I70, I92 | 4 | 1 | 0.00337 | 1. 24°50'15"N-121°30'50"E |
|  | Keelung | TSKL | H17, H34, H35, H37, H42, H44, H47, H50, H51 | 9 | 1 | 0.02622 | I6, I10, I25, I28, I30, I70, I98 | 7 | 1 | 0.00401 | 2. 25°07'30"N-121°34'35"E |
|  | **Touchien** | TC | H26, H30, H31, H33, H36, H44, H45, H48, H49 | 9 | 1 | 0.02789 | I2, I31, I44, I64, I79 | 5 | 1 | 0.00374 | 3. 24°43'10"N-121°15'05"E |
|  | **Chungkang** | CG | H01, H17(4) | 5 | 0.4 | 0.00080 | I13, I31, I85 | 3 | 1 | 0.00519 | 4. 24°40'50"N-121°00'55"E |
|  | **Houlung** | HLong | H01(4), H17 | 5 | 0.4 | 0.00080 | I5, I57, I70, I84, I9 | 5 | 1 | 0.00483 | 5. 24°28'00"N-120°58'10"E |
|  | **Ta-an** | TA | H01(5) | 5 | 0 | 0 | I46, I52, I87, I92 | 4 | 1 | 0.00398 | 6. 24°17'30"N-120°52'30"E |
|  | **Tachia** | TJ | H01(2), H17(3) | 5 | 0.6 | 0.00120 | I27, I33, I40 | 3 | 1 | 0.00312 | 7. 24°14'00"N-120°50'55"E |
|  | **Tatu** | TD |  |  |  |  |  |  |  |  |  |
|  | Dakeng Scenic Area | TDtk | H01(3), H17, H21, H29, H32, H38, H40 | 9 | 0.92 | 0.02322 | I11, I36, I38, I70, I80 | 5 | 1 | 0.00384 | 8. 24°11'05"N-120°53'00"E |
|  | Maolo | TDML | H01(4), H17 | 5 | 0.4 | 0.00080 | I4, I24, I51, I90, I100 | 5 | 1 | 0.00903 | 9. 24°02'10"N-120°38'50"E |
|  | **Choshui** | CS |  |  |  |  |  |  |  |  |  |
|  | Lien-Hua-Chih Area | CSlhc | H01(2), H14, H17, H20, H24, H25, H39, H46, | 9 | 0.97 | 0.02311 | I1, I16, I17, I50, I53, I70, I78 | 7 | 1 | 0.00267 | 10. 23°55'00"N-120°52'30"E |
|  | Sitou Forest Recreation Area | CSst | H01(3), H17, H20 | 5 | 0.7 | 0.00200 | I8, I9, I27, I89, I95 | 5 | 1 | 0.00623 | 11. 23°42'20"N-120°46'10"E |
|  | **Peikang** | PG | H01, H17(2) | 3 | 0.67 | 0.00133 | I76, I96 | 2 | 1 | 0.00779 | 12. 23°35'10"N-120°35'20"E |
|  | **Putzu** | PC | H05, H17 | 2 | 1 | 0.00400 | I67 | 1 |  |  | 13. 23°29'30"N-120°33'00"E |
|  | **Pachang** | PJ | H01, H06, H13, H22 | 4 | 1 | 0.00800 | I31, I37, I70 | 3 | 1 | 0.00415 | 14. 23°25'50"N-120°34'25"E |
|  | **Tsengwen** | TW |  |  |  |  |  |  |  |  |  |
|  | Tsengwen Reservoir Scenic Area | TWd | H08, H09 | 2 | 1 | 0.00400 | I29 | 1 |  |  | 15. 23°15'50"N-120°35'30"E |
|  | Tsailiao | TWTL | H01(3), H02, H17 | 5 | 0.7 | 0.00160 | I1(2), I15, I68 | 4 | 0.75 | 0.00234 | 16. 23°00'30"N-120°25'05"E |
|  | **Kaoping** | KP |  |  |  |  |  |  |  |  |  |
|  | Laonung | KPLN | H01, H17(2), H19 | 4 | 0.83 | 0.00200 | I18, I26, I55, I70 | 4 | 1 | 0.00467 | 17. 22°43'10"N-120°38'00"E |
|  | Chishan | KPCS | H01(5), H17 | 6 | 0.33 | 0.00067 | I12, I35, I58, I73, I93 | 5 | 1 | 0.00498 | 18. 22°58'20"N-120°29'30"E |
|  | **Linpian** | LB | H01, H17 | 2 | 1 | 0.00200 | I60, I62 | 2 | 1 | 0.00467 | 19. 22°32'45"N-120°39'30"E |
|  | **Fongkang** | FG | H01(3), H11, H17(2), H28, H41, H43 | 9 | 0.89 | 0.02644 | I42, I43, I45, I54, I59, I63, I69, I82 | 8 | 1 | 0.00729 | 20. 22°13'50"N-120°47'10"E |
|  | **Chihpen** | CB | H01, H17, H19 | 3 | 1 | 0.00267 | I88, I97 | 2 | 1 | 0.00727 | 21. 22°41'55"N-120°59'05"E |
|  | **Peinan** | PN | H01, H03, H16, H19, H27 | 5 | 1 | 0.00800 | I39, I47, I77 | 3 | 1 | 0.00831 | 22. 22°54'05"N-121°05'05"E |
|  | **Shiukuluan** | SKL | H01(4), H15 | 5 | 0.4 | 0.00160 | I21, I22, I38, I66, I75 | 5 | 1 | 0.00498 | 23. 23°11'55"N-121°16'50"E |
|  | **Hualien** | HLien | H01, H12 | 2 | 1 | 0.00200 | I41, I74 | 2 | 1 | 0.00623 | 24. 23°56'55"N-121°30'00"E |
|  | **Leewu** | LW | H01(2), H18 | 3 | 0.67 | 0.00133 | - | 0 |  |  | 25. 24°10'10"N-121°36'10"E |
|  | **Hoping** | HP | H01(3), H17(2) | 5 | 0.6 | 0.00120 | I61, I70, I72 | 3 | 1 | 0.00415 | 26. 24°21'45"N-121°44'05"E |
|  | **Chihming Waterfall** | CMW | H01(2), H10 | 3 | 0.67 | 0.00133 | I70 | 1 |  |  | 27. 24°20'20"N-121°45'50"E |
|  | **Nan-au** | NA | H01(4), H07 | 5 | 0.4 | 0.00080 | I14, I48, I49 | 3 | 1 | 0.00831 | 28. 24°28'35"N-121°44'45"E |
|  | **Xincheng** | XC | H01(5) | 5 | 0 | 0 | I65, I81, I91, I94 | 4 | 1 | 0.00872 | 29. 24°36'55"N-121°48'00"E |
|  | **Lanyang** | LY | H01, H04, H17(3) | 5 | 0.7 | 0.00200 | I1, I3, I19, I34, I55 | 5 | 1 | 0.00405 | 30. 24°41'50"N-121°36'55"E |
|  | **Derchihkou** | DCK | H01, H17(3), H23 | 5 | 0.7 | 0.00320 | I7, I16(2), I20, I86 | 5 | 0.8 | 0.00249 | 31. 24°49'30"N-121°45'00"E |
|  | **Shihmenkeng** | SMK | H01(4), H17 | 5 | 0.7 | 0.00080 | I1, I23, I70, I71, I83 | 5 | 1 | 0.00312 | 32. 25°17'30"N-121°34'20"E |
| *E. yayeyamana* | **Ishigaki jimma** | Ey | Ey01(2), Ey02, Ey03(2), Ey04 | 6 |  |  | Ey01, Ey02 | 2 |  |  | 24°22'53"N-124°12'00"E |
| *E. decorata* | **Ta Po Kau Country Park, Hong Kong** | *E. decorata* |  | 1 |  |  |  | 1 |  |  | 22°26'11"N-114°09'23"E |
| *E. impar* | **Malaysia peninsular** | *E. impar* |  | 0 |  |  |  | 1 |  |  | AJ746322 |
| *E. subcostalis* | **Gurah Recreaion Forest, Gurah, Sumatra** | *E. subcostalis* |  | 1 |  |  |  | 1 |  |  | 03°40'55"N-97°39'13"E |
| *E. refulgens* | **Mt. Isarog National Park, Luzon, Philippines** | *E. refulgens* |  | 1 |  |  |  | 1 |  |  | 13°39'47"N-123°19'56"E |
